# Ethyl 2-{3-[(2-chloro-1,3-thia­zol-5-yl)meth­yl]-4-nitro­imino-1,3,5-triazinan-1-yl}acetate

**DOI:** 10.1107/S1600536810018878

**Published:** 2010-05-26

**Authors:** Chuan-wen Sun, Jun Zhu, Jia Jin, Ding-rong Yang

**Affiliations:** a College of Life and Environmental Science, Shanghai Normal University, Shanghai 200234, People’s Republic of China

## Abstract

In the title compound, C_11_H_15_ClN_6_O_4_S, which belongs to the neonicotinoid class of insecticidally active heterocyclic compounds, the six-membered triazine ring adopts an opened envolope conformation. The planar nitro imine group [dihedral angle between nitro and imine groups = 1.07 (7)°] and the thia­zole ring are oriented at a dihedral angle of 69.62 (8)°. A classical intra­molecular N—H⋯O hydrogen bond is found in the mol­ecular structure. Moreover, one classical inter­molecular N—H⋯N and four non-classical C—H⋯O and C—H⋯N hydrogen bonds are also present in the crystal structure. Besides inter­molecular hydrogen bonds, the Cl atom forms an inter­molecular short contact [3.020 (2) Å] with one of the nitro O atoms.

## Related literature

For general background to neonicotinoid compounds and their application as insecticides, see: Kagabu (1996 ▶); Kagabu *et al.* (2005 ▶); Tian *et al.* (2007 ▶); Tomizawa *et al.* (2000 ▶); Tomizawa & Yamamoto (1993 ▶); Zhang *et al.* (2004 ▶). For halogen bonding, see: Riley & Merz (2007 ▶). For the synthesis of the title compound, see: Maienfisch *et al.* (2001 ▶).
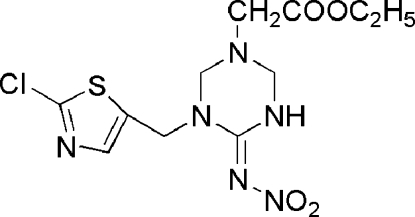


## Experimental

### 

#### Crystal data

C_11_H_15_ClN_6_O_4_S
*M* *_r_* = 362.81
Triclinic, 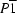

*a* = 8.5066 (6) Å
*b* = 9.1114 (7) Å
*c* = 10.9071 (8) Å
α = 100.488 (2)°
β = 98.416 (3)°
γ = 101.281 (3)°
*V* = 800.55 (10) Å^3^
*Z* = 2
Mo *K*α radiation
μ = 0.40 mm^−1^
*T* = 298 K
0.40 × 0.23 × 0.20 mm

#### Data collection

Bruker SMART APEX CCD area-detector diffractometer
Absorption correction: multi-scan (*SADABS*; Bruker, 2000 ▶) *T*_min_ = 0.857, *T*_max_ = 0.925
5350 measured reflections
3259 independent reflections
2670 reflections with *I* > 2σ(*I*)
*R*_int_ = 0.075

#### Refinement

*R*[*F* ^2^ > 2σ(*F* ^2^)] = 0.047
*wR*(*F* ^2^) = 0.128
*S* = 1.05
3259 reflections
212 parameters
9 restraints
H atoms treated by a mixture of independent and constrained refinement
Δρ_max_ = 0.31 e Å^−3^
Δρ_min_ = −0.26 e Å^−3^

### 

Data collection: *SMART* (Bruker, 2001 ▶); cell refinement: *SAINT* (Bruker, 2001 ▶); data reduction: *SAINT*; program(s) used to solve structure: *SHELXS97* (Sheldrick, 2008 ▶); program(s) used to refine structure: *SHELXL97* (Sheldrick, 2008 ▶); molecular graphics: *PLATON* (Spek, 2009 ▶); software used to prepare material for publication: *PLATON*.

## Supplementary Material

Crystal structure: contains datablocks I, global. DOI: 10.1107/S1600536810018878/rk2201sup1.cif

Structure factors: contains datablocks I. DOI: 10.1107/S1600536810018878/rk2201Isup2.hkl

Additional supplementary materials:  crystallographic information; 3D view; checkCIF report

## Figures and Tables

**Table 1:** Hydrogen-bond geometry (Å, °)

*D*—H⋯*A*	*D*—H	H⋯*A*	*D*⋯*A*	*D*—H⋯*A*
N3—H3*A*⋯N6^i^	0.79 (2)	2.55 (2)	3.133 (2)	132 (2)
N3—H3*A*⋯O3	0.79 (2)	1.98 (2)	2.570 (2)	131 (2)
C3—H3*C*⋯O3^ii^	0.97	2.57	3.191 (3)	122
C5—H5*A*⋯N1^iii^	0.97	2.61	3.449 (3)	144
C6—H6*B*⋯O4^iv^	0.97	2.56	3.478 (3)	159
C10—H10⋯O1^v^	0.93	2.45	3.278 (3)	149

Symmetry codes: (i) 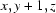; (ii) 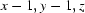; (iii) 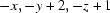; (iv) 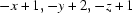; (v) 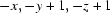.

**Fig. 1.**
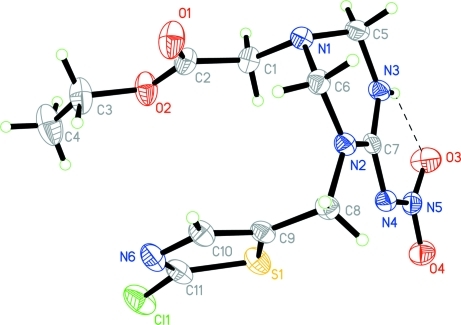
The molecular structure of title compound with the atom numbering scheme. Displacement ellipsoids are drawn at 50% probability level. H atoms are presented as a small spheres of arbitrary radius. The intramolecular H–bond is marked by dashed line.

## supplementary crystallographic information

### Comment

Neonicotinoid compounds (Tomizawa & Yamamoto, 1993; Kagabu, 1996; Tomizawa *et al.*, 2000) have received much attention on their applications as insecticide (Zhang *et al.*, 2004; Kagabu *et al.*, 2005; Tian *et al.*, 2007). We report here the molecular and crystal structures of the title compound.

The molecular structure of title compound, C_11_H_15_ClN_6_O_4_S, is depicted on Fig.1. The triazine moiety exhibits an opened envolope conformation with N1 out of the envolope plan defined by C5, N3, C7, N2 and C6. The dihedral angle between the thiazole ring and triazine envolope plan is 75.63 (7)°. The planar nitroimine–group and the thiazole ring are oriented the dihedral angle of 69.62 (8)°. The large discrepancy between (O4—N5···N4 115.65 (17)°) and (O3—N5···N4 123.84 (16)°) bond angles attributes to the hydrogen bond effect on O4 and O3 - look the Table 1. Another interesting structure feature that should be mentioned is that the bond lengths (C7—N2 1.340 (2)Å) and (C7—N3 1.329 (2)Å) are between the standard (C—N 1.47Å) single bond and (C═N 1.26Å) double bond, clearly showing the conjugated effect of the nirtoimine. The C7—N4 bond length is as long as to 1.357 (2)Å, due to being linked with a strong electron–attracting nitro–group. Moreover, except intermolecular hydrogen bonding, the crystal structure is further stabilized by the so–called halogen bonding (Riley & Merz, 2007), due to short intermolecular contact of Cl1—O4^i^ with a distance of 3.020 (2)Å and an angle close to 180° (C11—Cl1···O4^i^ 178.1 (1)°). Symmetry code: (i) 1-*x*,1-*y*,-*z*).

### Experimental

The title compound was prepared by the literature method (Maienfisch *et al.*, (2001). It was purified by silica gel chromatography using ethyl acetate and petroleum ether in the ratio of 1:1, as the flush to afford. This compound was obtained as white crystals, yield 46.7%, ^1^H NMR(CDCl_3_, 400 Hz): 9.51 (1*H*, s, NH), 7.44 (1*H*, s, thiazole—H), 4.61 (2*H*,s,CH_2_—thiazole), 4.48–4.49(4*H*, d, *J* = 5.2 Hz, triazine—4H), 4.21–4.15 (2*H*, m, OCH_2_), 3.32 (2*H*, s, CHC═O) 1.29–1.26 (3*H*, t, *J* = 7.2 Hz, CH_3_); IR(potassium bromide, cm^-1^) 3288(N—H), 3000 (thiazole), 1730 (C═O) 1587 (C═N), 1398 (NO_2_), 1224 (C—O—C), 1105 (C—N), Anal. calcd for C_11_H_15_ClN_6_O_4_S: C 36.42, H 4.17, N 23.16; found C 36.40, H 4.23, N 23.19. ESI–MS m/z: 363.8.

### Refinement

H atoms bonded to C atoms were positioned geometrically [C—H = 0.93Å (aromatic), 0.97Å (methylene) and 0.96Å (methyl)] and refined in riding modes with *U*_iso_(H) = 1.2*U*_eq_(C) for aromatic and methylene; *U*_iso_(H) = 1.5*U*~eq~(C) for methyl. H atom bonded to N atom was found from Fourier difference maps and refined with the constraint *U*_iso_(H) = 1.2*U*_eq_(N), but coordinates refined freely.

### Crystal data

**Table:**

C_11_H_15_ClN_6_O_4_S	*Z* = 2
*M**_r_* = 362.81	*F*(000) = 376
Triclinic, *P*1	*D*_x_ = 1.505 Mg m^−^^3^
Hall symbol: -P 1	Melting point: 449 K
*a* = 8.5066 (6) Å	Mo *K*α radiation, λ = 0.71073 Å
*b* = 9.1114 (7) Å	Cell parameters from 2512 reflections
*c* = 10.9071 (8) Å	θ = 2.3–28.2°
α = 100.488 (2)°	µ = 0.40 mm^−^^1^
β = 98.416 (3)°	*T* = 298 K
γ = 101.281 (3)°	Block, colourless
*V* = 800.55 (10) Å^3^	0.40 × 0.23 × 0.20 mm

### Data collection

**Table:**

Bruker SMART APEX CCD area-detector diffractometer	3259 independent reflections
Radiation source: fine focus sealed Siemens Mo tube	2670 reflections with *I* > 2σ(*I*)
graphite	*R*_int_ = 0.075
Detector resolution: 1.57 × 0.49 mm pixels mm^-1^	θ_max_ = 26.5°, θ_min_ = 1.9°
0.3° wide ω scans	*h* = −10→9
Absorption correction: multi-scan (*SADABS*; Bruker, 2000)	*k* = −10→11
*T*_min_ = 0.857, *T*_max_ = 0.925	*l* = −13→12
5350 measured reflections	

### Refinement

**Table:**

Refinement on *F*^2^	Primary atom site location: structure-invariant direct methods
Least-squares matrix: full	Secondary atom site location: difference Fourier map
*R*[*F*^2^ > 2σ(*F*^2^)] = 0.047	Hydrogen site location: inferred from neighbouring sites
*wR*(*F*^2^) = 0.128	H atoms treated by a mixture of independent and constrained refinement
*S* = 1.05	*w* = 1/[σ^2^(*F*_o_^2^) + (0.0598*P*)^2^ + 0.0779*P*] where *P* = (*F*_o_^2^ + 2*F*_c_^2^)/3
3259 reflections	(Δ/σ)_max_ < 0.001
212 parameters	Δρ_max_ = 0.31 e Å^−^^3^
9 restraints	Δρ_min_ = −0.26 e Å^−^^3^

### Special details

**Table:**

Geometry. All s.u.'s (except the s.u. in the dihedral angle between two l.s. planes) are estimated using the full covariance matrix. The cell s.u.'s are taken into account individually in the estimation of s.u.'s in distances, angles and torsion angles; correlations between s.u.'s in cell parameters are only used when they are defined by crystal symmetry. An approximate (isotropic) treatment of cell s.u.'s is used for estimating s.u.'s involving l.s. planes.

### Fractional atomic coordinates and isotropic or equivalent isotropic displacement parameters (Å^2^)

**Table:**

	*x*	*y*	*z*	*U*_iso_*/*U*_eq_	
C1	−0.0095 (3)	0.7149 (2)	0.26414 (19)	0.0444 (5)	
H1A	0.0880	0.7030	0.2309	0.053*	
H1B	−0.0751	0.7574	0.2054	0.053*	
C2	−0.1049 (2)	0.5590 (2)	0.2702 (2)	0.0454 (5)	
C3	−0.2096 (4)	0.3054 (3)	0.1509 (3)	0.0701 (7)	
H3C	−0.3189	0.3031	0.1674	0.084*	
H3B	−0.1526	0.2605	0.2128	0.084*	
C4	−0.2173 (5)	0.2185 (3)	0.0216 (3)	0.0943 (11)	
H4A	−0.2647	0.2692	−0.0391	0.141*	
H4B	−0.2831	0.1169	0.0105	0.141*	
H4C	−0.1091	0.2126	0.0092	0.141*	
C5	0.1108 (3)	0.9754 (2)	0.3748 (2)	0.0485 (5)	
H5A	0.1225	1.0469	0.4549	0.058*	
H5B	0.0397	1.0059	0.3108	0.058*	
C6	0.1510 (2)	0.7783 (2)	0.47718 (18)	0.0405 (4)	
H6A	0.1079	0.6734	0.4825	0.049*	
H6B	0.1619	0.8435	0.5603	0.049*	
C7	0.3648 (2)	0.88803 (19)	0.36999 (17)	0.0337 (4)	
C8	0.4008 (2)	0.6726 (2)	0.46817 (19)	0.0408 (4)	
H8A	0.3775	0.6440	0.5462	0.049*	
H8B	0.5174	0.7146	0.4805	0.049*	
C9	0.3537 (2)	0.5325 (2)	0.36264 (18)	0.0399 (4)	
C10	0.2576 (3)	0.3953 (2)	0.3607 (2)	0.0468 (5)	
H10	0.2113	0.3768	0.4302	0.056*	
C11	0.3072 (3)	0.3403 (2)	0.1709 (2)	0.0527 (6)	
Cl1	0.30601 (10)	0.23735 (8)	0.02224 (6)	0.0779 (3)	
N1	0.03775 (19)	0.82249 (18)	0.38653 (16)	0.0408 (4)	
N2	0.31341 (19)	0.79068 (17)	0.44198 (15)	0.0367 (4)	
N3	0.2716 (2)	0.98200 (18)	0.33909 (17)	0.0393 (4)	
H3A	0.306 (3)	1.039 (3)	0.297 (2)	0.047*	
N4	0.50886 (19)	0.87424 (18)	0.33519 (16)	0.0402 (4)	
N5	0.5757 (2)	0.97002 (19)	0.26754 (16)	0.0430 (4)	
N6	0.2308 (2)	0.28347 (19)	0.25118 (19)	0.0544 (5)	
O1	−0.1559 (2)	0.52487 (19)	0.36034 (15)	0.0659 (5)	
O2	−0.1227 (2)	0.46316 (17)	0.16030 (15)	0.0614 (4)	
O3	0.5158 (2)	1.0752 (2)	0.23700 (19)	0.0671 (5)	
O4	0.70673 (19)	0.95104 (19)	0.23875 (17)	0.0607 (4)	
S1	0.41711 (8)	0.52681 (6)	0.21876 (5)	0.0532 (2)	

### Atomic displacement parameters (Å^2^)

**Table:**

	*U*^11^	*U*^22^	*U*^33^	*U*^12^	*U*^13^	*U*^23^
C1	0.0449 (11)	0.0457 (11)	0.0389 (10)	0.0019 (9)	0.0037 (9)	0.0122 (9)
C2	0.0434 (11)	0.0495 (12)	0.0386 (11)	0.0005 (9)	0.0066 (9)	0.0095 (9)
C3	0.0903 (19)	0.0483 (12)	0.0592 (14)	−0.0107 (12)	0.0183 (13)	0.0055 (11)
C4	0.144 (3)	0.0561 (15)	0.0660 (17)	−0.0045 (17)	0.0236 (19)	−0.0031 (12)
C5	0.0451 (11)	0.0377 (10)	0.0671 (14)	0.0144 (8)	0.0191 (10)	0.0106 (10)
C6	0.0430 (10)	0.0429 (10)	0.0331 (10)	0.0067 (8)	0.0087 (8)	0.0037 (8)
C7	0.0379 (9)	0.0256 (8)	0.0336 (9)	0.0057 (7)	0.0040 (7)	−0.0001 (7)
C8	0.0473 (11)	0.0370 (10)	0.0389 (10)	0.0130 (8)	0.0030 (8)	0.0106 (8)
C9	0.0479 (11)	0.0363 (10)	0.0398 (10)	0.0176 (8)	0.0065 (8)	0.0118 (8)
C10	0.0590 (13)	0.0391 (11)	0.0460 (12)	0.0163 (9)	0.0105 (10)	0.0122 (9)
C11	0.0731 (15)	0.0436 (11)	0.0427 (11)	0.0274 (10)	0.0013 (11)	0.0048 (10)
Cl1	0.1162 (6)	0.0694 (4)	0.0455 (3)	0.0420 (4)	0.0031 (3)	−0.0065 (3)
N1	0.0398 (9)	0.0382 (8)	0.0447 (9)	0.0101 (7)	0.0090 (7)	0.0074 (7)
N2	0.0405 (8)	0.0318 (8)	0.0385 (8)	0.0098 (6)	0.0071 (7)	0.0080 (7)
N3	0.0416 (9)	0.0306 (8)	0.0499 (10)	0.0112 (6)	0.0133 (7)	0.0124 (7)
N4	0.0375 (8)	0.0364 (8)	0.0468 (9)	0.0082 (6)	0.0106 (7)	0.0075 (7)
N5	0.0401 (9)	0.0421 (9)	0.0407 (9)	0.0023 (7)	0.0079 (7)	0.0009 (7)
N6	0.0704 (12)	0.0356 (9)	0.0545 (11)	0.0156 (8)	0.0039 (10)	0.0053 (8)
O1	0.0766 (11)	0.0650 (10)	0.0453 (9)	−0.0146 (8)	0.0207 (8)	0.0100 (8)
O2	0.0806 (11)	0.0484 (9)	0.0455 (9)	−0.0096 (8)	0.0202 (8)	0.0055 (7)
O3	0.0612 (10)	0.0647 (11)	0.0929 (14)	0.0183 (8)	0.0302 (9)	0.0448 (10)
O4	0.0468 (9)	0.0706 (11)	0.0654 (10)	0.0116 (7)	0.0249 (8)	0.0070 (8)
S1	0.0731 (4)	0.0452 (3)	0.0456 (3)	0.0181 (3)	0.0187 (3)	0.0098 (2)

### Geometric parameters (Å, °)

**Table:**

C1—N1	1.455 (3)	C6—H6B	0.9700
C1—C2	1.510 (3)	C7—N3	1.329 (2)
C1—H1A	0.9700	C7—N2	1.340 (2)
C1—H1B	0.9700	C7—N4	1.357 (2)
C2—O1	1.198 (2)	C8—N2	1.468 (2)
C2—O2	1.318 (3)	C8—C9	1.498 (3)
C3—O2	1.460 (3)	C8—H8A	0.9700
C3—C4	1.472 (4)	C8—H8B	0.9700
C3—H3C	0.9700	C9—C10	1.348 (3)
C3—H3B	0.9700	C9—S1	1.728 (2)
C4—H4A	0.9600	C10—N6	1.380 (3)
C4—H4B	0.9600	C10—H10	0.9300
C4—H4C	0.9600	C11—N6	1.287 (3)
C5—N1	1.447 (2)	C11—S1	1.717 (2)
C5—N3	1.469 (3)	C11—Cl1	1.717 (2)
C5—H5A	0.9700	N3—H3A	0.79 (2)
C5—H5B	0.9700	N4—N5	1.339 (2)
C6—N1	1.444 (3)	N5—O4	1.237 (2)
C6—N2	1.476 (2)	N5—O3	1.243 (2)
C6—H6A	0.9700		
			
N1—C1—C2	113.48 (16)	N3—C7—N4	127.89 (17)
N1—C1—H1A	108.9	N2—C7—N4	113.62 (16)
C2—C1—H1A	108.9	N2—C8—C9	112.19 (16)
N1—C1—H1B	108.9	N2—C8—H8A	109.2
C2—C1—H1B	108.9	C9—C8—H8A	109.2
H1A—C1—H1B	107.7	N2—C8—H8B	109.2
O1—C2—O2	124.32 (19)	C9—C8—H8B	109.2
O1—C2—C1	126.2 (2)	H8A—C8—H8B	107.9
O2—C2—C1	109.47 (17)	C10—C9—C8	127.86 (19)
O2—C3—C4	108.0 (2)	C10—C9—S1	109.13 (15)
O2—C3—H3C	110.1	C8—C9—S1	123.00 (14)
C4—C3—H3C	110.1	C9—C10—N6	117.05 (19)
O2—C3—H3B	110.1	C9—C10—H10	121.5
C4—C3—H3B	110.1	N6—C10—H10	121.5
H3C—C3—H3B	108.4	N6—C11—S1	117.24 (17)
C3—C4—H4A	109.5	N6—C11—Cl1	123.01 (18)
C3—C4—H4B	109.5	S1—C11—Cl1	119.74 (15)
H4A—C4—H4B	109.5	C6—N1—C5	107.68 (16)
C3—C4—H4C	109.5	C6—N1—C1	113.41 (16)
H4A—C4—H4C	109.5	C5—N1—C1	111.93 (16)
H4B—C4—H4C	109.5	C7—N2—C8	121.33 (16)
N1—C5—N3	111.07 (15)	C7—N2—C6	120.93 (15)
N1—C5—H5A	109.4	C8—N2—C6	116.72 (15)
N3—C5—H5A	109.4	C7—N3—C5	122.00 (17)
N1—C5—H5B	109.4	C7—N3—H3A	115.8 (16)
N3—C5—H5B	109.4	C5—N3—H3A	122.2 (16)
H5A—C5—H5B	108.0	N5—N4—C7	118.95 (16)
N1—C6—N2	112.02 (15)	O4—N5—O3	120.48 (17)
N1—C6—H6A	109.2	O4—N5—N4	115.65 (17)
N2—C6—H6A	109.2	O3—N5—N4	123.84 (16)
N1—C6—H6B	109.2	C11—N6—C10	108.32 (18)
N2—C6—H6B	109.2	C2—O2—C3	116.56 (17)
H6A—C6—H6B	107.9	C11—S1—C9	88.24 (10)
N3—C7—N2	118.48 (17)		
			
N1—C1—C2—O1	7.9 (3)	N1—C6—N2—C8	−143.03 (17)
N1—C1—C2—O2	−171.21 (18)	N2—C7—N3—C5	−3.8 (3)
N2—C8—C9—C10	−105.2 (2)	N4—C7—N3—C5	174.62 (18)
N2—C8—C9—S1	73.8 (2)	N1—C5—N3—C7	−28.0 (3)
C8—C9—C10—N6	179.47 (18)	N3—C7—N4—N5	4.3 (3)
S1—C9—C10—N6	0.3 (2)	N2—C7—N4—N5	−177.22 (16)
N2—C6—N1—C5	−55.1 (2)	C7—N4—N5—O4	−179.85 (17)
N2—C6—N1—C1	69.28 (19)	C7—N4—N5—O3	2.1 (3)
N3—C5—N1—C6	56.0 (2)	S1—C11—N6—C10	0.8 (2)
N3—C5—N1—C1	−69.3 (2)	Cl1—C11—N6—C10	179.62 (16)
C2—C1—N1—C6	65.1 (2)	C9—C10—N6—C11	−0.7 (3)
C2—C1—N1—C5	−172.82 (16)	O1—C2—O2—C3	−0.5 (4)
N3—C7—N2—C8	173.29 (16)	C1—C2—O2—C3	178.7 (2)
N4—C7—N2—C8	−5.4 (2)	C4—C3—O2—C2	179.5 (2)
N3—C7—N2—C6	5.2 (3)	N6—C11—S1—C9	−0.57 (19)
N4—C7—N2—C6	−173.49 (16)	Cl1—C11—S1—C9	−179.41 (14)
C9—C8—N2—C7	−82.8 (2)	C10—C9—S1—C11	0.11 (16)
C9—C8—N2—C6	85.8 (2)	C8—C9—S1—C11	−179.09 (17)
N1—C6—N2—C7	25.6 (2)		

### Hydrogen-bond geometry (Å, °)

**Table:**

*D*—H···*A*	*D*—H	H···*A*	*D*···*A*	*D*—H···*A*
N3—H3A···N6^i^	0.79 (2)	2.55 (2)	3.133 (2)	132 (2)
N3—H3A···O3	0.79 (2)	1.98 (2)	2.570 (2)	131 (2)
C3—H3C···O3^ii^	0.97	2.57	3.191 (3)	122
C5—H5A···N1^iii^	0.97	2.61	3.449 (3)	144
C6—H6B···O4^iv^	0.97	2.56	3.478 (3)	159
C10—H10···O1^v^	0.93	2.45	3.278 (3)	149

Symmetry codes: (i) *x*, *y*+1, *z*; (ii) *x*−1, *y*−1, *z*; (iii) −*x*, −*y*+2, −*z*+1; (iv) −*x*+1, −*y*+2, −*z*+1; (v) −*x*, −*y*+1, −*z*+1.
